# Enhancing Product Quality, Nutrition, Antioxidant Capacity, and Sensory Quality of Chicken Sausages by Replacing Fats with *Agaricus bisporus* and Soybean Oil

**DOI:** 10.3390/foods14132296

**Published:** 2025-06-28

**Authors:** Haijuan Nan, Haixu Zhou, Tetiana M. Stepanova, Zongshuai Zhu, Bo Li

**Affiliations:** 1 School of Food Science, Henan Institute of Science and Technology, No. 655 Hua Lan Street, Xinxiang 453003, China; nanhaijuan1@163.com (H.N.); haixuzhou_zh@126.com (H.Z.); libo@hist.edu.cn (B.L.); 2 Henan Engineering Research Center of Fruit and Vegetable Processing and Quality Safety Control, No. 655 Hua Lan Street, Xinxiang 453003, China; 3 School of Food Technology, Sumy National Agrarian University, 160 Herasima Kondratieva Street, 40021 Sumy, Ukraine; tetiana.stepanova@snau.edu.ua

**Keywords:** *Agaricus bisporus*, soybean oil, fat substitutes, chicken sausage, product quality, nutrition, antioxidant capacity, sensory quality

## Abstract

There are growing health concerns regarding high-fat meat products. This study systematically evaluated the quality of reformulated chicken sausages through progressive substitution (30%, 60%, and 90%) of traditional pork-back fat with an *Agaricus bisporus*–soybean oil complex. The 60% substitution optimized texture, fatty acids, and sensory properties: hardness increased from 4332.38 N (control) to 5810.04 N, and chewiness from 3048.55 N to 3896.93 N. Linoleic acid (C18:2n6) rose from 13.00 to 32.81 g/100 g and α-linolenic acid (C18:3n3) from 0.60 to 3.05 g/100 g, improving the PUFA/SFA ratio from 0.40 to 1.15). Sensory scores (flavor/taste/overall) increased from 6.0/5.1/6.6 to 7.2/5.6/7.4. After 35-day storage, TBARS values (0.161, 0.147, 0.126 mg/100 g for 30%/60%/90% groups) remained below the control (0.232 mg/100 g). Meanwhile, the reduced-fat sausages exhibited a deeper, less saturated red hue. Scanning electron microscopy (SEM) revealed an enhanced network structure in the sausage matrix. The reformulated sausages maintained essential product characteristics such as cooking yield, moisture retention, protein content, and amino acid profile while achieving a 9.5–16.1% reduction in energy value. These findings collectively demonstrate that the *A. bisporus*–soybean oil complex effectively enhances the product quality, nutrition, antioxidant capacity, and sensory quality of reduced-fat chicken sausages, demonstrating this plant-based composite as a promising functional ingredient for developing healthier meat products.

## 1. Introduction

Chicken serves as a nutritionally significant protein source (averaging 20–23% protein content) in modern diets, providing essential amino acids crucial for musculoskeletal maintenance and organ function. Its lipid profile is predominantly composed of cardioprotective unsaturated fatty acids while containing micronutrients including B vitamins (B_6_, B_12_), iron, and zinc that support immune function, neurodevelopment, and hematopoiesis [1]. Processed chicken products, particularly sausages, offer comparable nutritional value with enhanced consumption convenience.

Chicken sausages embody a nutritional dichotomy: animal fats play an indispensable role in maintaining product stability, flavor, and texture of emulsified meat products [2]. However, epidemiological evidence links excessive consumption of such lipid-rich processed meats to elevated risks of metabolic disorders, including obesity and hypertension, primarily due to their high energy density and saturated fatty acid profile [3,4]. This dilemma has spurred research into lipid-reduced formulations [5], yet fat reduction often compromises sensory quality. Conventional fat replacers, including polysaccharides [6], dietary fibers [7], pre-emulsions [8], protein-based substitutes [9], and liquid vegetable oils [10,11,12], are manufactured using established techniques and have gained widespread use in various meat products. While these substitutes effectively reduce animal fat content and improve water-holding capacity and textural properties, they generally fail to replicate the flavor, sensory attributes, and nutritional profile of animal fats. Furthermore, the fatty acid composition of the meat product remains largely unchanged (except when vegetable oils are directly added). Consequently, researchers are actively exploring novel fat replacer systems, such as oil gels [13], emulsion gels [14], and Pickering emulsion gels [15]. In contrast to conventional options, these novel systems modify the lipid phase itself, enabling superior simulation of animal fat mouthfeel. However, their development presents new challenges: **(i)** they can compromise gel structure integrity, reduce water retention, diminish flavor intensity, cause color fading, and induce lipid oxidation; and (ii) their commercial application is currently hampered by low consumer acceptance. Consequently, current substitution attempts have shown limited success in replicating the functional properties of animal fats [16], highlighting the need for more effective alternatives that balance nutritional benefits with product quality.

*Agaricus bisporus*, a globally cultivated edible mushroom, demonstrates significant potential as a functional ingredient in meat products due to its high protein content, dietary fiber, and bioactive polysaccharides (mannose, trehalose) that enhance textural properties [17]. Its rich amino acid profile, particularly essential amino acids, further contributes to flavor development in processed meats [18]. Concurrently, soybean oil, a nutritionally valuable lipid source, contains cardioprotective unsaturated fatty acids (linoleic and linolenic acids), vitamin E, and phytosterols that improve product stability while addressing health concerns associated with animal fats [19]. Despite these individual advantages, no prior research has systematically evaluated the integrated application of *Agaricus bisporus* and soybean oil (ABSO) as a fat-replacement system in poultry sausages. To address both technological challenges and health considerations in meat product reformulation, this study therefore investigated the synergistic effects of ABSO on the product quality, nutrition, antioxidant capacity, and sensory quality of reduced-fat chicken sausages [20]. The innovations of this study lie in proposing a novel method using *Agaricus bisporus* and soybean oil as a composite fat substitute and verifying the applicability of this composite fat substitute in reduced-fat chicken sausages.

## 2. Materials and Methods

### 2.1. Material

All experimental materials, including soybean oil, chilled chicken breast, fresh *Agaricus bisporus* (Ab), pork back fat, sodium chloride (NaCl), white pepper, sucrose, chicken essence, potato starch, and 25 mm collagen casings (Spanish origin), were commercially sourced from a regional retail chain (Hualan Supermarket, Xinxiang, China). A local food manufacturer in China provided sodium tripolyphosphate. Other chemical reagents met analytical standards.

### 2.2. Agaricus bisporus (Ab) Powder Preparation and Pretreatment of Chicken and Pork Back Fat

Ab powder was prepared by referring to the previous method with slight modifications [21]. Fresh Ab mushroom was cleaned, sliced into 5 mm thick slices, and dried in a drying oven (DHG-9013A, Shanghai Yiheng Scientific Instrument Co., Ltd., Shanghai Municipality, China) for 8 h at 45 °C. The dried mushrooms (7% moisture) were then pulverized followed by particle size fractionation using standard sieve classification (160 μm aperture). The resultant homogenized Ab particulates were hermetically sealed in low-density polyethylene (LDPE) barrier packaging for subsequent experiments. Chicken and fat were preprocessed as previously reported [17]. The remaining visible connective tissue was removed from the meat and fat. Using a grinder (MM-12, Guangdong, China) equipped with a 6 mm diameter aperture configuration, chicken and fat were ground separately. The minced meat and fat were then placed in nylon/polyethylene bags, sealed, and kept at a temperature of −20 °C until they were consumed within four weeks.

### 2.3. Manufacture of Sausages

Ab powder was premixed with soybean oil (1:2 *w*/*w*) 24 h before sausage preparation. Following previously described standard procedures [17], meat batters were processed using a commercial grinder (Joyoung S2-A808) according to the formula in Table 1. Thawed minced meat (12 h, 4 °C) underwent three-stage emulsification in a bowl cutter (JR65-G220, Supor Co., Ltd., Hangzhou, Zhejiang, China): initial chopping (1500 rpm, 60 s) with NaCl, tripolyphosphate and 33% ice-water (3 min rest); secondary mixing (1500 rpm, 120 s) with remaining ingredients and additional 33% ice-water (3 min rest); final emulsification (3000 rpm, 60 s) with residual ice-water, maintaining the temperature <10 °C. The batter was stuffed into collagen casings (10 cm length) and thermally processed (baked for 30 min at 60 °C, then cooked for 30 min at 80 °C). Post-cooking, the sausages were vacuum-packed, underwent thermal treatment of the packed sausages (15 s, 80 °C), were chilled in an ice slurry, and stored at 4 °C. Each batch produced four treatments of chicken sausage, and three separate batches were created.

### 2.4. Composition and Energy Value

Proximate composition analysis (moisture—950.46, ash—923.03, crude protein—992.15, and total lipids—996.06) was performed according to AOAC Official Methods of Analysis [22]. Amino acid profiling and fatty acid quantification were conducted in strict adherence to the Chinese National Standard [23,24]. Three samples were used for each treatment in triplicate for all analyses. The following formulas were used to determine the total amount of carbohydrates and energy values [25].

(1) Total carbohydrates (g/100g)=100−(mmoisture+mprotein+mfat+mash)

(2) Energy value(kcal/g)=4×(mprotein+mcarbohydrate)+9mfat

### 2.5. Cooking Yield (CY), Color and pH

The cooking yield (CY) was determined by measuring the weight ratio of cooked to raw sausage after 24 h storage at 4 °C, during which exudate was collected and removed. Each formulation was analyzed in triplicate.

Chromatic characterization employed standardized cylindrical specimens (20 mm height) sectioned from the sausage. According to earlier reports [17], a CR-400 chromameter (Minolta, Chiyoda-ku, Tokyo, Japan) was positioned flush against freshly prepared cross-sections for quantitative colorimetric analysis. Chromatic parameters (L*, a*, b*) were quantified through quintuplicate measurements per formulation with subsequent arithmetic mean calculation. △E* indicates the overall color difference between the experimental group and the control group. Chroma (C) indicates color vividness. The Hue value (H°) represents the direction of the color tone. The following Formulas (3)–(5) were used to calculate △E*, C, and H° values, respectively.

(3) $$△E*=\sqrt{{\left(L^{*}-L_{CK}^{*}\right)}^{2}+{\left(a^{*}-a_{CK}^{*}\right)}^{2}+{\left(b^{*}-b_{CK}^{*}\right)}^{2}}$$

(4) $$C=\sqrt{{{(a}^{*})}^{2}+({b^{*})}^{2}}$$

(5) $$H{}^{\circ}=arctan(\frac{b^{*}}{a^{*}})$$

The pH of the sausage was determined according to previously reported methods [26] with some modifications. Ab powder (10 g) was homogenized with 0.1 M KCl (100 g) using an IKA T25 digital homogenizer (IKA-Werke, Staufen im Breisgau, Germany) at 8000 rpm for 60 s. Triplicate pH measurements of the filtrate were performed for each formulation using a calibrated Mettler-Toledo 320 pH meter (Mettler-Toledo GmbH, Greifensee, Switzerland), with values reported as arithmetic means.

### 2.6. Texture Profile Analysis (TPA)

Sausages were analyzed within 24 h post-production. Samples were prepared by removing the sausages from vacuum packaging, gently blotting surfaces with filter paper, discarding end segments (1 cm), and using the central portion for testing. The central segments were axially sectioned into 25 mm diameter × 20 mm height cylinders using a sharp stainless-steel blade (rinsed with hot water and dried between cuts to prevent sample adhesion). These cylinders served as texture analysis specimens. The textural properties of the sausage were measured using the previously reported measurement parameters [17,27]. Texture profile analysis (TPA) was conducted in quintuplicate using a TA.XT Plus texture analyzer (Stable Micro Systems, Godalming, UK) at room temperature. Instrumental protocols included P36R probe calibration (30 mm working distance), dual-cycle compression at 50% strain, and sequential velocity parameters: 2 mm/s (pre-test/test phases), 5 mm/s (post-test), with 5 s inter-cycle intervals. Quantified parameters included hardness (N), elasticity, viscosity and chewiness (N·mm).

### 2.7. Lipid Oxidation (TBARS)

Lipid oxidation in sausages was quantified via a thiobarbituric acid reactive substance (TBARS) assay following previously reported procedures [28]. TBARS analysis was conducted every 5 days during 35-day storage. In detail, 5 g minced samples were homogenized with 20 mL distilled water for 3 min, firstly. After adding 25 mL of trichloroacetic acid solution, the mixture underwent a ten-minute centrifugation at 2000 rpm after remaining at room temperature for one hour. The supernatant was diluted to 50 mL with distilled water, and 5 mL of this was thoroughly mixed with an equal volume ratio of 0.02 mol/L 2-thiobarbituric acid solution. After 20 min of incubation at 95 °C, the mixture underwent a series of five minutes of cooling at 0 °C. Then, chloroform (10 mL) was incorporated, following by a 10 min centrifugation at 2000 rpm. Absorbance quantification was performed at λ = 532 nm against a deionized water reference blank using UV-Vis spectroscopy. The following formulas were used to determine the value of TBARS.

(6) TBARS value(mg/100 g sausage)=7.8×A532

### 2.8. Microstructure

The microstructure of sausage was determined based on previous reports [17]. The sausages were first cut into cubes of size 3 × 3 × 3 mm^3^; then, cubic chicken sausage specimens underwent primary fixation in 2.5% glutaraldehyde-phosphate buffer solution (PBS, pH 6.8) at 4 °C for 24 h. Following fixation, triple washing cycles were conducted using 0.1 mol/L PBS (pH 6.8, 15 min per wash). Gradual dehydration was achieved through an ethanol series (50%, 60%, 70%, 80%, 90%—15 min each; 100%—10 min × 3), followed by lipid extraction in chloroform (1 h). Subsequent solvent exchange involved sequential immersion in: (1) 1:1 (*v*/*v*) ethanol-tert-butyl alcohol mixture, and (2) pure tert-butyl alcohol (15 min each). Lyophilization was carried out at 45 °C for 5 h under vacuum. Prepared specimens were affixed to bronze stubs, sputter-coated with gold powder, and the microstructure was then observed by scanning electron microscopy (Quanta 200, FEI CO., Hillsboro, OR, USA). Finally, the most representative ones were selected from the many photos taken.

### 2.9. Sensory Evaluation

Sensory evaluation was performed by previously reported methods [29]. The sausages were kept in a 4 °C refrigerator overnight before being brought to room temperature, and then sensory evaluations were conducted. Sensory evaluation was completed by twenty faculty and student volunteers (gender-balanced, 18–60 years old) from Henan Institute of Science and Technology, Xinxiang, China [29]. Informed consent for participation was obtained from all subjects involved in the study. All participants received protocol-specific training before evaluation. Each batch of samples was sliced to the same size, placed on a white plate, and randomly numbered with three digits. To examine the sample’s sensory quality, the assessors scored the sausages on a nine-point happiness scale based on color, flavor, texture, and general acceptability (extremely like = 9, neither like nor dislike = 5, extremely dislike = 2).

### 2.10. Statistical Analysis

Statistical analyses were performed using SPSS Statistics 20.0 (IBM) with a one-way ANOVA and Duncan’s multiple range test (*p* < 0.05) for inter-group comparisons. Quantitative data are presented as mean ± SD following parametric assumptions.

## 3. Results and Discussion

### 3.1. Properties and Composition of Ab Powder and Sausage

**Figure 1:**
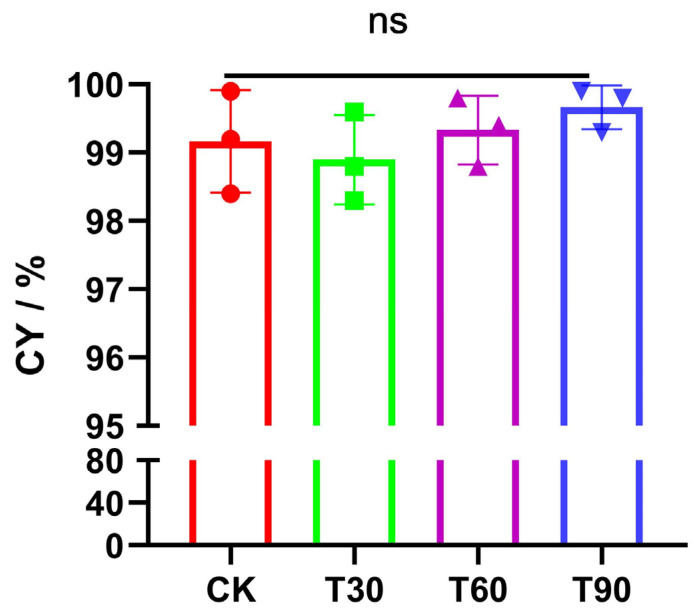
Effects of replacing lipid with ABSO on the cooking yield of sausages (g/100 g). Experimental groups (CK, T30, T60, T90) corresponded to 0%, 30%, 60%, and 90% lipid substitution in sausage formulations. ‘ns’ indicates no significant intergroup differences (*p* > 0.05) within experimental groups.

Proximate analysis (Table 2 and Table 3) revealed the composition of mushroom powder and sausage. Sausage formulations exhibited significant (*p <* 0.05) lipid reduction with progressive fat substitution, accompanied by proportional ash–carbohydrate accretion. This inverse nutritional relationship mirrors the mycoprotein’s inherent low-fat, high-ash–carbohydrate profile. Notably, these findings align with established strategies augmenting meat matrices through non-animal protein supplementation, as documented in seaweed and spirulina applications [30,31]. When compared to CK, the protein level of the T90 dramatically decreased (*p* < 0.05). This is likely due to the fact that soybean oil, which makes up two-thirds of the Ab premix, contains almost no protein, but pork back fat has 2.4% protein [32]. Protein levels in T30 and T60 formulations remained stable (*p* > 0.05), likely reflecting adequate compensation from Ab-derived proteins despite partial lipid substitution. Moisture retention showed no intergroup variance (*p* > 0.05), indicating preserved sausage juiciness independent of ABSO-mediated fat replacement (Figure 1). Notably, T60 and T90 exhibited elevated ash and carbohydrate concentrations versus CK and T30 (*p* < 0.05), attributable to Ab powder’s inherent inorganic ions and polysaccharide profile (Table 2). Compared with the control group, the fat and energy values (T30–T90) exhibited statistically significant reductions of 9.8–30.5% and 9.5–16.1%, respectively (*p* < 0.05), demonstrating the efficacy of ABSO as a functional ingredient for low-calorie food production.

### 3.2. Amino Acids (AA) Profile

Amino acid (AA) profiles exerted critical influences on the nutritional quality and sensory characteristics of sausage products. Quantitative analysis revealed dose-dependent enhancement of total AA (TAA), essential AA (EAA), and non-essential AA (NEAA) concentrations in ABSO-formulated sausages versus control (CK) (Table 4). Significant increases (*p* < 0.05) were particularly observed in T30’s TAA and EAA and T90’s NEAA fractions while maintaining EAA/NEAA ratios within 0.98–1.06 across all formulations. Dominant AAs comprising leucine, lysine, aspartate, glutamate, and arginine mirrored the mycoprotein’s intrinsic AA spectrum (Table 2). Notably, umami-enhancing glutamate/aspartate enrichment mechanistically explained flavor intensification through ABSO incorporation [33]. This stability in AA composition during fat substitution aligns with documented preservation patterns in fungal-supplemented meat analogs [18].

### 3.3. Fatty Acid Profile of Sausage

Fatty acid composition serves as a critical determinant of sausage nutritional quality. Lipidomic profiling enables systematic quantification of saturated (SFA), unsaturated (UFA), monounsaturated (MUFA), and polyunsaturated (PUFA) fatty acids, establishing robust metrics for nutritional evaluation and dietary compliance (Table 5). In ABSO-modified formulations, progressive substitution induced dose-dependent reductions in total SFA (excluding C20:0/C22:0) and MUFA (*p* < 0.05), primarily driven by attenuated C16:1, C17:1, C20:1, and C18:1n9c levels across T30–T90 groups. Notably, C18:1n9c decreased from 45.51 to 26.71 g/100 g (CK→T90), while PUFA exhibited inverse concentration dependence (*p* < 0.05). These modulations align with lipid restructuring patterns observed in plant oil-fortified meat analogs [34], though divergent outcomes arose from the different fatty acid compositions of olive oil-based systems [34]. ABSO supplementation induced dose-dependent alterations in fatty acid profiles, with C18:2n6 (linoleic acid) and C18:3n3 (α-linolenic acid) exhibiting significant elevations (*p* < 0.05), contrasting the marked depletion of C20:2. Notably, reformulated sausages demonstrated progressive enrichment of these essential fatty acids (EFAs): C18:2n6 concentrations escalated from 13.00 g/100 g (CK) to 22.35, 32.81, and 47.03 g/100 g in T30–T90 groups, respectively, while C18:3n3 levels surged from 0.60 g/100 g (CK) to 1.82–4.78 g/100 g. This lipid restructuring aligns with the inherent composition of soybean oil (42.92 g/100 g C18:2n6; 0.87 g/100 g C18:3n3) [35], the primary EFA source requiring dietary intake. Crucially, T60 achieved optimal PUFA/SFA (0.8–1) and ω-6/ω-3 (5–10) ratios compliant with WHO nutritional guidelines [36], establishing its superiority in lipid nutritional quality.

### 3.4. Cooking Yield (CY)

Controlling cooking loss was crucial in sausage production, as it impacted product yield, texture, and flavor, thus directly influencing product quality. The CY values of all the sausages were close to 100%, and there was no significant difference between them (*p* > 0.05) due to the high dietary fiber level in Ab powder [37], which absorbed water and oil, thus preventing cooking loss due to decreased fat content. It has been reported that pea pod fibers improved the cooking yield of low-fat goat meat batter [38], which was not in line with the present findings. The absence of significant improvement in the sausages’ CY values can be attributed to their near-saturation levels (100%), approaching the theoretical maximum yield under the applied processing conditions. The exceptional water- and oil-absorption capacities demonstrated by Ab powder result from synergistic effects of physical adsorption (porous structure), chemical bonding, and swelling encapsulation (gel formation). Primarily, the freeze-dried mushroom cell walls form a three-dimensional porous network structure, where high-specific-surface-area pores rapidly immobilize and retain moisture and lipids in sausage products through capillary action and surface tension. Secondarily, abundant hydroxyl groups on β-glucan molecular chains establish hydrogen bonding interactions with water molecules, while non-polar groups (e.g., acetyl -OCOCH_3_ and methyl -CH_3_) in cell wall polysaccharides form van der Waals complexes with aliphatic chains of triglycerides [39]. Thirdly, water-soluble dietary fibers such as β-glucans undergo hydration-induced swelling to generate a viscous gel matrix, which physically entraps fluid molecules and restricts their diffusion through steric hindrance effects [40,41].

### 3.5. Color

Color parameters (L*, a*, b*) represent critical quality determinants in sausages, profoundly influencing consumer acceptance. As detailed in Table 6, ABSO substitution induced chromatic modifications characterized by progressive L* reduction (brightness attenuation) and concurrent a* and b* elevation (redness/yellowness intensification) (*p* < 0.05). The inverse correlation between ABSO dosage and luminosity (*p* < 0.05) paralleled enhanced redness, attributable to Ab powder’s inherent chromatic properties (Table 2). While b* values exhibited dosage-dependent decline, all treatment groups maintained significantly higher yellowness than CK (*p* < 0.05). Total color deviation (ΔE*) demonstrated proportional escalation with ABSO incorporation, aligning with previous findings on Ab-mediated chromatic alterations in poultry matrices [17]. From a technological perspective, this natural pigment enrichment suggests potential for synthetic colorant reduction in processed meats, pending validation through consumer sensory trials. Chroma (C), indicating color vividness, increases with higher values. ABSO fat substitution consistently elevated sausage chroma compared to the control, suggesting enhanced color intensity despite the gradual decline in C-value with increasing substitution levels (*p* < 0.05). 0° Hue value corresponds to red, and 90° Hue value corresponds to yellow. The progressive decrease in Hue values from 88.27° (CK) to 80.77° (T90) (*p* > 0.05) indicates a tonal shift toward the red spectrum with increasing fat replacement levels. The possible reason for this shift is that ABSO altered the protein–fat matrix, affecting the light scattering path, particularly when the ABSOs themselves contain pigments. Combined with chroma (C-value) decline data, this actually manifests as a dark red tone with reduced saturation. Sensory evaluation is required to assess consumer acceptance of such alterations.

### 3.6. TPA

The texture of sausages serves as a critical quality indicator, interacting dynamically with other attributes like flavor and color. Key textural parameters—including hardness (resistance to deformation), springiness (ability to recover shape), cohesiveness (internal binding strength), and chewiness (energy required for mastication)—directly determine sensory perception during consumption. Notably, optimal textural properties enhance flavor delivery by effectively retaining and gradually releasing aroma compounds during chewing. This synchronization between structural breakdown and flavor release allows consumers to fully experience the product’s sensory profile. Such texture–flavor interactions highlight the importance of texture engineering in developing premium meat products. Textural profiling via instrumental texture analysis (TA-XT Plus) revealed dose-dependent elevations in hardness (42.5–61.8 N) and chewiness (29.9–40.0 N·mm) across reformulated sausages (T60–T90), with significant intergroup differences (*p* < 0.05) relative to control formulations (CK) (Table 7). While springiness maintained stability (0.93–0.94), marked cohesiveness attenuation (0.76→0.70) occurred at maximum supplementation (T90). These textural enhancements align with the documented effects of fungal β-glucans on meat matrix restructuring [37], particularly through ionic modulation of myofibrillar protein gelation dynamics. The divalent cation load (Ca^2+^/Mg^2+^) in Ab powder facilitated salt-soluble protein extraction, promoting three-dimensional network formation [42,43,44]. However, suboptimal ionic strength (T90) likely induced excessive protein charge screening, disrupting hydrophobic interactions critical for viscoelastic maintenance [45]. Optimal textural parameters in T60 corresponded with moderate ionic conditions, demonstrating the precision required in functional ingredient dosage for meat product engineering.

### 3.7. TBARS Value

TBARS serve as critical oxidative biomarkers in sausage quality assessment, quantifying lipid peroxidation dynamics that directly correlate with nutrient degradation, organoleptic profile deterioration, and shelf-life truncation. As is shown in Table 8, both the control (CK) and T30 groups showed a continuous increase in TBARS values during storage, with peak levels observed on day 25. During the first five days of storage, the sausage with ABSO showed higher TBARS values than the CK. Thermally induced Maillard reaction derivatives and protein degradation products in *Ab* powder may exhibit absorbance at 532 nm, potentially interfering with thiobarbituric acid reactive substances (TBARS) quantification. The TBARS values of T60 and T90 decreased from 0 to 15 days, then increased and peaked at 25 days of storage. The decrease could be due to pigment degradation in Ab mushrooms. The increase could be connected to fat oxidation in the sausage. The TBARS values for T90 were lower than those for T60 during the late storage period, suggesting that the increasing Ab content enhanced the antioxidant ability of the sausage against fat. This is likely due to the large amounts of phenols and flavonoids in Ab mushrooms, which are potent antioxidants [37]. Current research found that ABSO reduced the lipid oxidation in the sausage, which was consistent with previous reports that dried Ab inhibited lipid oxidation in cooked beef [46]. Contrary findings exist regarding Ab powder’s antioxidant efficacy, with a recent trial demonstrating non-significant inhibition of lipid peroxidation in processed meat systems (*p* > 0.05) [18]. This functional discrepancy likely originates from dynamic fluctuations in the mushroom’s bioactive compounds, particularly β-glucans and ergothioneine concentrations, which exhibit marked sensitivity to cultivation parameters, harvest maturity stages, and post-processing dehydration protocols [47]. The fluctuating TBARS values during refrigerated storage (around 4 °C) suggest that fat oxidation played only a minor role in spoiling the chicken sausages. This is likely because the fat in the sausages was tightly surrounded by a protective layer of soluble proteins. This protein “shield” not only blocked oxygen from reaching the fat (preventing oxidation) but also repelled microbes that break down fats, thanks to the protein layer’s natural negative charge. Essentially, the proteins acted as both a physical barrier and a microbial deterrent, keeping the fat stable over time.

### 3.8. Microstructure

**Figure 2:**
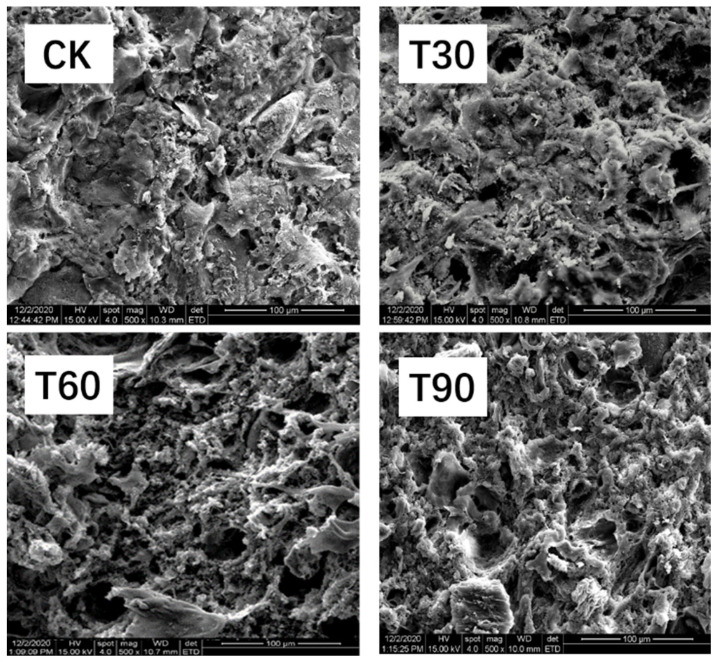
Effects of replacing lipid with ABSO on microstructure of sausages. (Scale bar: 100 μm, Mag: 500×). Experimental groups (CK, T30, T60, T90) corresponded to 0%, 30%, 60%, and 90% lipid substitution in sausage formulations.

Scanning electron microscopy (SEM), a widely utilized analytical technique in material characterization, was employed to investigate the surface morphology, structural composition, and topographical features of experimental samples. These microstructural parameters hold particular significance in evaluating the textural properties and quality attributes of processed meat products. In the current investigation, SEM analysis was systematically conducted to examine the architectural modifications in chicken sausage matrices following the incorporation of ABSO. As demonstrated in Figure 2, microstructural alterations were observed with ABSO supplementation. Control samples (CK) exhibited characteristic granular surfaces with irregular porosity and heterogeneous matrix distribution. Conversely, ABSO-treated groups displayed various structural modifications, manifesting as enhanced matrix homogeneity, reduced void spaces, and formation of cohesive interfacial junctions in comparison with the control samples (CK). This enhancement of the network structure was intrinsically linked to the improved textural properties of sausages [48]. Within this study, sausages incorporating ABSO demonstrated enhanced textural characteristics, thereby aligning with the observations from the microstructural analysis. Meanwhile, these findings align with previous rheological measurements and texture profile analyses, confirming the structure–function relationship in modified meat protein systems [17]. The observed phenomenon may be primarily attributed to three synergistic mechanisms governing protein–polysaccharide interactions. Firstly, the macromolecular architecture of Ab mushroom dietary fiber facilitated intermolecular crosslinking through steric entrapment and hydrogen bonding, thereby reinforcing the three-dimensional protein gel network formation. Secondly, Ab mushroom dietary fiber exhibited exceptional hydrophilicity due to its abundant hydroxyl and carboxyl groups [40]. This pronounced hydration-swelling behavior enabled effective occupation of interstitial spaces within the protein matrix, concurrently decreasing mean porosity and increasing gel density. Thirdly, the surface-active functional groups (-NH_2_, -COOH) in Ab mushroom dietary fiber exhibited enhanced oil-binding capacity, enabling efficient ABSO dispersion through interfacial tension reduction. This optimized emulsification process decreased myofibrillar protein allocation for lipid stabilization, redirecting critical sarcoplasmic proteins toward continuous phase gelation. The synergistic effects of these mechanisms—network reinforcement, spatial confinement, and phase redistribution—collectively enhanced textural parameters [41,49,50,51].

### 3.9. Sensory Evalution

**Figure 3:**
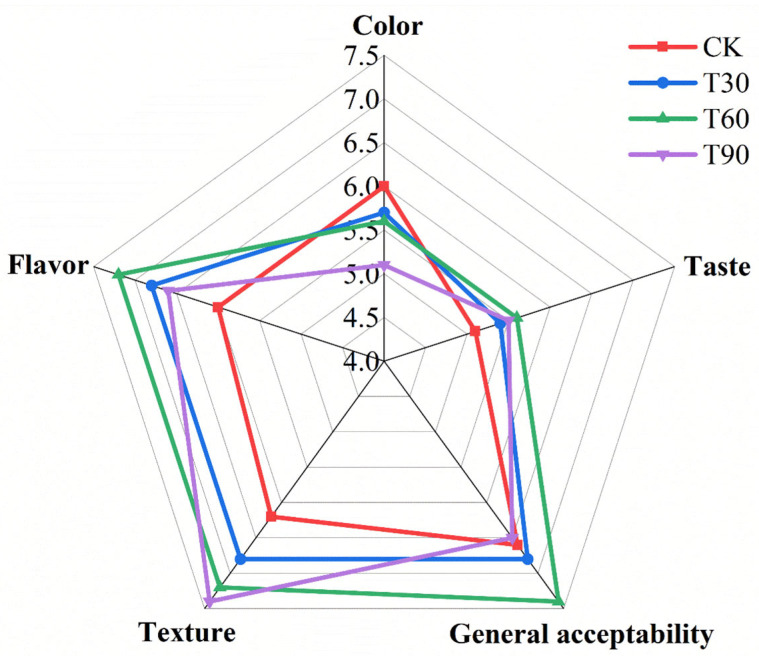
Effects of replacing lipid with ABSO on sensory score of sausages. Experimental groups (CK, T30, T60, T90) corresponded to 0%, 30%, 60%, and 90% lipid substitution in sausage formulations.

A comprehensive sensory evaluation of the sausage was performed to quantify five critical sensory attributes: taste profile, color, flavor, texture, and general acceptability. As is shown in Figure 3, the addition of ABSO increased the flavor, general acceptability, texture, and taste scores of the sausages while decreasing the color scores; however, the color scores of sausages with ABSO were all greater than 5.0, indicating that consumers could accept the color change caused by ABSO. T60 received the highest taste, flavor, and general acceptability scores, indicating that ABSO should be added in appropriate amounts. In sum, consumers showed greater interest in the chicken sausages with ABSO in place of 60% fat.

## 4. Conclusions

This study for the first time applied an ABSO composite fat substitute in reduced-fat chicken sausages. The results showed that while ABSO incorporation maintained cooking yield (*p* > 0.05), it notably enhanced nutritional profiles by reducing fat content and energy value while elevating ash and carbohydrate levels in chicken sausages (*p* < 0.05). Partial fat replacement (60–90%) improved texture (hardness and chewiness) and the ratio of ω-6/ω-3. Partial fat replacement (30–60%) improved essential amino acid composition and the ratio of PUFA/SFA and delayed lipid oxidation. Optimal sensory acceptance was observed at 60% substitution. ABSO-induced color shifts manifested as decreased L* values and increased a* and b* values. The reduced-fat sausage presents as a dark red hue with reduced saturation. The application results of this innovative method demonstrated that 60% fat replacement with ABSO achieves balanced product quality, nutrition, antioxidant capacity and consumer preference, thus making up for the deficiencies of the reported fat substitutes. ABSO is a promising functional fat substitute. Future studies should elucidate molecular interactions between soybean oil components and mushroom-derived polysaccharides with myofibrillar proteins to optimize fat mimetic functionality.

## Figures and Tables

**Table 1:** Formula of chicken sausage based on 100 g of ground batter.

Raw Material/Ingredients	Formula (g)			
	CK	T30	T60	T90
Chicken batter				
Chicken	60	60	60	60
Pork back fat	20	14	8	2
Ab mushroom	0	2	4	6
Soybean oil	0	4	8	12
Ice water	20	20	20	20
Others				
Salt	1.4	1.4	1.4	1.4
Sodium tripolyphosphate	0.3	0.3	0.3	0.3
Chicken essence	0.1	0.1	0.1	0.1
Sugar	0.65	0.65	0.65	0.65
White pepper	0.15	0.15	0.15	0.15
Potato starch	5	5	5	5
The total sum	107.6	107.6	107.6	107.6

**Table 2:** The physicochemical properties of Ab powder.

Composition (%)		Color and pH	
Moisture	6.96 ± 0.11	L*	51.26 ± 0.49
Ash	10.15 ± 0.34	a*	1.91 ± 0.09
Protein	22.14 ± 0.53	b*	14.70 ± 0.13
Fat	2.53 ± 0.05	pH	6.53 ± 0.05
Carbohydrate	58.22 ± 0.73		
Essential amino acid (%)		Non-essential Amino acid (%)	
Val	1.19 ± 0.10	ASP	1.89 ± 0.11
Met	0.27 ± 0.01	Glu	5.02 ± 0.24
Ile	1.02 ± 0.03	Ser	0.83 ± 0.02
Leu	1.44 ± 0.09	Gly	1.11 ± 0.08
Phe	0.97 ± 0.02	Pro	1.01 ± 0.05
His	0.41 ± 0.01	Ala	1.79 ± 0.02
Lys	1.21 ± 0.10	Cyst	0.06 ± 0.01
Thr	1.03 ± 0.08	Tyr	0.77 ± 0.02
Arg	0.98 ± 0.02		

n = 1. Data represent physicochemical properties analysis of a single batch; no inter-group comparisons were made.

**Table 3:** Proximate composition of the sausage.

Items	CK	T30	T60	T90
Moisture (g/100 g)	61.06 ± 1.32 ^a^	64.58 ± 2.22 ^a^	61.85 ± 1.18 ^a^	62.71 ± 1.25 ^a^
Ash (g/100 g)	0.73 ± 0.07 ^c^	0.76 ± 0.05 ^c^	1.54 ± 0.01 ^b^	1.92 ± 0.06 ^a^
Protein (g/100 g)	15.38 ± 1.40 ^a^	14.36 ± 0.99 ^a^	12.51 ± 1.48 ^ab^	11.90 ± 0.94 ^b^
Fat (g/100 g)	18.56 ± 0.31 ^a^	16.74 ± 0.24 ^b^	14.80 ± 0.34 ^c^	12.90 ± 0.21 ^d^
Carbohydrate (g/100 g)	4.27 ± 0.37 ^c^	3.56 ± 0.35 ^c^	9.30 ± 0.34 ^b^	10.57 ± 0.35 ^a^
Energy (kcal/100 g)	245.64 ± 9.87 ^a^	222.34 ± 7.52 ^b^	220.44 ± 10.34 ^b^	205.98 ± 7.05 ^b^

n = 3. Experimental groups (CK, T30, T60, T90) denoted 0%, 30%, 60%, and 90% lipid substitution in sausage formulations, respectively. Superscript letters within rows reflect statistically significant intergroup variations (*p* < 0.05).

**Table 4:** Effects of replacing lipid with ABSO on amino acid profile of sausages (g/100 g).

Amino Acids	CK	T30	T60	T90
Essential				
Val	0.77 ± 0.01 ^b^	0.83 ± 0.02 ^a^	0.82 ± 0.01 ^a^	0.81 ± 0.01 ^a^
Met	0.34 ± 0.01 ^c^	0.43 ± 0.02 ^a^	0.38 ± 0.02 ^b^	0.41 ± 0.02 ^ab^
Ile	0.69 ± 0.02 ^c^	0.84 ± 0.03 ^a^	0.77 ± 0.01 ^b^	0.75 ± 0.01 ^b^
Leu	1.10 ± 0.05 ^a^	1.32 ± 0.21 ^a^	1.21 ± 0.10 ^a^	1.19 ± 0.08 ^a^
Phe	0.61 ± 0.02 ^c^	0.72 ± 0.02 ^a^	0.66 ± 0.01 ^b^	0.65 ± 0.01 ^b^
His	0.34 ± 0.01 ^a^	0.35 ± 0.02 ^a^	0.34 ± 0.01 ^a^	0.35 ± 0.02 ^a^
Lys	1.18 ± 0.08 ^b^	1.37 ± 0.09 ^a^	1.26 ± 0.07 ^ab^	1.32 ± 0.08 ^ab^
The	0.69 ± 0.02 ^b^	0.77 ± 0.01 ^a^	0.73 ± 0.01 ^a^	0.74 ± 0.02 ^a^
Arg	1.04 ± 0.08 ^a^	1.02 ± 0.05 ^a^	1.02 ± 0.04 ^a^	1.03 ± 0.03 ^a^
Non-essential				
ASP	1.23 ± 0.06 ^b^	1.38 ± 0.03 ^a^	1.34 ± 0.05 ^ab^	1.38 ± 0.04 ^a^
Glu	2.13 ± 0.15 ^a^	2.29 ± 0.05 ^a^	2.33 ± 0.06 ^a^	2.35 ± 0.07 ^a^
Ser	0.41 ± 0.01 ^c^	0.49 ± 0.01 ^a^	0.45 ± 0.02 ^b^	0.50 ± 0.01 ^a^
Gly	0.71 ± 0.01 ^d^	0.82 ± 0.01 ^b^	0.76 ± 0.02 ^c^	0.95 ± 0.01 ^a^
Pro	0.55 ± 0.01 ^d^	0.64 ± 0.01 ^b^	0.59 ± 0.01 ^c^	0.69 ± 0.01 ^a^
Ala	0.80 ± 0.02 ^c^	0.91 ± 0.01 ^a^	0.85 ± 0.02 ^b^	0.90 ± 0.03 ^ab^
Cyst	0.10 ± 0.01 ^b^	0.13 ± 0.01 ^a^	0.11 ± 0.01 ^ab^	0.11 ± 0.01 ^ab^
Tyr	0.51 ± 0.02 ^ab^	0.53 ± 0.01 ^a^	0.49 ± 0.02 ^b^	0.52 ± 0.02 ^ab^
TAA	13.19 ± 0.59 ^b^	14.85 ± 0.61 ^a^	14.12 ± 0.49 ^ab^	14.63 ± 0.48 ^ab^
∑EAA	6.76 ± 0.30 ^b^	7.65 ± 0.47 ^a^	7.19 ± 0.28 ^ab^	7.25 ± 0.28 ^ab^
∑NEAA	6.44 ± 0.29 ^b^	7.19 ± 0.14 ^ab^	6.92 ± 0.21 ^b^	7.40 ± 0.20 ^a^
EAA/NEAA	1.05 ± 0.02 ^b^	1.06 ± 0.01 ^a^	1.04 ± 0.02 ^ab^	0.98 ± 0.02 ^b^

Experimental groups (CK, T30, T60, T90) denoted 0%, 30%, 60%, and 90% lipid substitution in sausage formulations, respectively. Superscript letters within rows reflect statistically significant intergroup variations (*p* < 0.05). Abbreviations: TAA (total amino acids), EAA (essential amino acids), NEAA (non-essential amino acids).

**Table 5:** Effects of replacing lipid with ABSO on fatty acid of sausages (g/100 g).

Fatty Acid	CK	T30	T60	T90
C14:0	1.10 ± 0.23 ^a^	0.92 ± 0.14 ^a^	0.61 ± 0.12 ^b^	0.26 ± 0.21 ^c^
C15:0	0.05 ± 0.00 ^a^	0.05 ± 0.00 ^a^	-	-
C16:0	23.70 ± 2.45 ^a^	20.81 ± 0.91 ^a^	17.73 ± 1.02 ^b^	13.54 ± 1.23 ^c^
C17:0	0.27 ± 0.02 ^a^	0.24 ± 0.01 ^a^	0.19 ± 0.01 ^b^	-
C18:0	10.91 ± 2.34 ^a^	9.43 ± 1.96 ^ab^	7.52 ± 1.55 ^ab^	5.17 ± 1.02 ^b^
C20:0	-	-	0.44 ± 0.01 ^b^	0.58 ± 0.02 ^a^
C22:0	0.10 ± 0.02 ^d^	0.19 ± 0.02 ^c^	0.27 ± 0.04 ^b^	0.37 ± 0.03 ^a^
∑SFA	36.13 ± 2.39 ^a^	31.64 ± 2.08 ^b^	26.76 ± 2.34 ^c^	19.92 ± 2.06 ^d^
C16:1	2.20 ± 0.54 ^a^	1.93 ± 0.21 ^a^	1.29 ± 0.11 ^b^	0.59 ± 0.35 ^c^
C17:1	0.29 ± 0.01 ^a^	0.26 ± 0.02 ^a^	0.17 ± 0.04 ^b^	0.09 ± 0.00 ^c^
C20:1	0.98 ± 0.07 ^a^	0.85 ± 0.16 ^a^	0.59 ± 0.09 ^b^	0.36 ± 0.05 ^c^
C18:1n9t	-	-	-	-
C18:1n9c	45.51 ± 3.15 ^a^	39.81 ± 2.01 ^b^	34.63 ± 2.16 ^c^	26.71 ± 3.04 ^d^
C22:1n9	0.32 ± 0.03 ^a^	0.35 ± 0.02 ^a^	0.31 ± 0.04 ^a^	0.30 ± 0.01 ^a^
C24:1	0.10 ± 0.00 ^a^	0.10 ± 0.00 ^a^	0.09 ± 0.01 ^a^	0.09 ± 0.01 ^a^
∑MUFA	49.41 ± 3.12 ^a^	43.3 ± 2.45 ^b^	37.09 ± 2.46 ^c^	28.14 ± 2.37 ^d^
C18:2n6	13.00 ± 1.04 ^d^	22.35 ± 2.36 ^c^	32.81 ± 2.41 ^b^	47.03 ± 2.17 ^a^
C18:3n6	0.27 ± 0.08 ^a^	0.38 ± 0.04 ^a^	-	-
C18:3n3	0.60 ± 0.04 ^d^	1.82 ± 0.11 ^c^	3.05 ± 0.63 ^b^	4.78 ± 1.04 ^a^
C20:2	0.53 ± 0.08 ^a^	0.45 ± 0.07 ^a^	0.29 ± 0.02 ^b^	0.13 ± 0.01 ^c^
C20:3n3	0.06 ± 0.01 ^a^	0.06 ± 0.00 ^a^	-	-
∑PUFA	14.47 ± 2.31 ^d^	25.06 ± 3.05 ^c^	36.15 ± 3.14 ^b^	51.94 ± 3.48 ^a^
PUFA/SFA	0.40 ± 0.02 ^d^	0.79 ± 0.07 ^c^	1.15 ± 0.09 ^b^	2.61 ± 1.01 ^a^
PUFA/MUFA	0.29 ± 0.02 ^d^	0.58 ± 0.11 ^c^	0.97 ± 0.14 ^b^	1.85 ± 0.19 ^a^
MUFA/SFA	1.37 ± 0.03 ^a^	1.37 ± 0.09 ^a^	1.39 ± 0.08 ^a^	1.41 ± 0.14 ^a^
(MUFA+PUFA)/SFA	1.77 ± 0.09 ^d^	2.16 ± 0.11 ^c^	2.74 ± 0.24 ^b^	4.02 ± 0.85 ^a^
Omega6/Omega3	21.67	12.28	10.76	9.84

Experimental groups (CK, T30, T60, T90) corresponded to 0%, 30%, 60%, and 90% lipid substitution in sausage formulations. Fatty acid nomenclature: SFA (saturated), PUFA (polyunsaturated), MUFA (monounsaturated). Superscript letters indicate significant intergroup differences (*p* < 0.05) within analytical rows.

**Table 6:** Effects of replacing lipid with ABSO on the color of sausages.

Items	CK	T30	T60	T90
L*	86.78 ± 0.06 ^a^	75.52 ± 0.23 ^b^	68.42 ± 0.39 ^c^	64.20 ± 0.15 ^d^
a*	0.43 ± 0.01 ^d^	1.24 ± 0.02 ^c^	2.40 ± 0.02 ^b^	2.65 ± 0.03 ^a^
b*	14.25 ± 0.15 ^d^	17.47 ± 0.09 ^a^	16.82 ± 0.08 ^b^	16.29 ± 0.02 ^c^
△E*	-	11.74 ± 0.18 ^c^	18.64 ± 0.34 ^b^	22.78 ± 0.16 ^a^
C	14.26 ± 0.15 ^d^	17.52 ± 0.09 ^a^	16.99 ± 0.08 ^b^	16.50 ± 0.04 ^c^
H°	88.27 ± 15.00 ^a^	85.94 ± 4.50 ^a^	81.87 ± 4.00 ^a^	80.77 ± 0.67 ^a^

Experimental groups (CK, T30, T60, T90) corresponded to 0%, 30%, 60%, and 90% lipid substitution in sausage formulations. Superscript letters indicate significant intergroup differences (*p* < 0.05) within analytical rows.

**Table 7:** Effects of replacing lipid with ABSO on TPA of sausages.

TPA	CK	T30	T60	T90
Hardness/N	42.5 ± 1.9 ^b^	43.3 ± 2.9 ^b^	57.0 ± 5.8 ^a^	61.8 ± 3.2 ^a^
Springiness	0.93 ± 0.00 ^a^	0.93 ± 0.00 ^a^	0.94 ± 0.01 ^a^	0.93 ± 0.01 ^a^
Cohesiveness	0.76 ± 0.00 ^a^	0.72 ± 0.02 ^b^	0.74 ± 0.02 ^ab^	0.70 ± 0.02 ^b^
Chewiness/N·mm	29.9 ± 1.0 ^b^	29.7 ± 0.8 ^b^	38.2 ± 1.5 ^a^	40.0 ± 0.7 ^a^

Experimental groups (CK, T30, T60, T90) corresponded to 0%, 30%, 60%, and 90% lipid substitution in sausage formulations. Superscript letters indicate significant intergroup differences (*p* < 0.05) within analytical rows.

**Table 8:** Effects of replacing lipid with ABSO on TBARS value of sausages (mg/100 g).

Day (d)	CK	T30	T60	T90
1	0.101 ± 0.001 ^d,D^	0.148 ± 0.001 ^c,E^	0.202 ± 0.001 ^b,C^	0.275 ± 0.001 ^a,A^
5	0.147 ± 0.004 ^d,C^	0.156 ± 0.001 ^c,D^	0.185 ± 0.003 ^b,D^	0.261 ± 0.006 ^a,B^
10	0.217 ± 0.013 ^a,B^	0.169 ± 0.006 ^b,C^	0.174 ± 0.003 ^b,E^	0.227 ± 0.003 ^a,C^
15	0.291 ± 0.003 ^a,B^	0.181 ± 0.007 ^b,B,C^	0.159 ± 0.004 ^c,F^	0.150 ± 0.005 ^c,E^
20	0.295 ± 0.001 ^a,B^	0.189 ± 0.001 ^bc,B^	0.235 ± 0.006 ^b,B^	0.181 ± 0.001 ^c,D^
25	0.328 ± 0.006 ^a,A^	0.224 ± 0.003 ^c,A^	0.247 ± 0.005 ^b,A^	0.223 ± 0.002 ^c,C^
30	0.298 ± 0.006 ^a,B^	0.210 ± 0.013 ^b,A^	0.159 ± 0.006 ^c,F^	0.102 ± 0.008 ^d,G^
35	0.232 ± 0.002 ^a,C^	0.161 ± 0.002 ^b,C^	0.147 ± 0.003 ^c,G^	0.126 ± 0.001 ^d,F^

Experimental groups (CK, T30, T60, T90) corresponded to 0%, 30%, 60%, and 90% lipid substitution in sausage formulations. Superscript lowercase (a, b, c, d) and uppercase letters (A, B, C, D, E, F, G) indicate statistically significant intergroup differences (*p* < 0.05) within the same analytical rows and the same column, respectively.

## Data Availability

The original contributions presented in the study are included in the article, further inquiries can be directed to the corresponding author.
